# Fit of Three-Unit Posterior Fixed Dental Prostheses Made from Tetragonal Zirconia Polycrystal by 3D Printing and Milling

**DOI:** 10.3390/ma19030597

**Published:** 2026-02-03

**Authors:** Jana Kostunov, Jannis Crocoll, Sebastian Hetzler, Peter Rammelsberg, Jonas Zeiß, Andreas Zenthöfer, Stefan Rues

**Affiliations:** Department of Prosthodontics, Medical Faculty, Heidelberg University, 69120 Heidelberg, Germany; jannis.crocoll@stud.uni-heidelberg.de (J.C.); sebastian.hetzler@med.uni-heidelberg.de (S.H.); peter.rammelsberg@med.uni-heidelberg.de (P.R.); jonas.zeiss@med.uni-heidelberg.de (J.Z.); andreas.zenthoefer@med.uni-heidelberg.de (A.Z.); stefan.rues@med.uni-heidelberg.de (S.R.)

**Keywords:** zirconia, 3D zirconia printing, three-unit FDP, marginal fit, internal fit

## Abstract

(1) Objective: To compare the marginal and internal fit of 3D-printed and milled three-unit fixed dental prostheses (FDPs) made from tetragonal zirconia polycrystal (3Y-TZP). (2) Methods: Three-unit FDPs were designed for a typodont maxillary model with crown preparation for the second premolar and second molar. Nominal cement gap widths were set to 30 µm at the margins and 80 µm internally. A total of 40 FDPs (*n* = 10/group) differing in wall thickness (w = 0.6/1.0 mm) and support structures (with/without a stiffening frame) were fabricated from 3Y-TZP by 3D printing. A total of 10 milled FDPs with w = 0.6 mm served as a control group. After adhesive cementation on the respective replicated maxillary models, FDPs were sectioned and the cement gap dimension was assessed with a digital microscope. The marginal and internal fit found for the different test groups were compared using non-parametric tests. (3) Results: The best marginal fit—qualified by median/maximum marginal gap width—was given for milled FDPs (79/127 µm vertical; 85/171 µm tangential), whereas the marginal fit of 3D-printed FDPs with w = 0.6 mm and regular support structures was the worst (144/284 µm vertical; 107/198 µm tangential). Use of an additional support frame improved the marginal fit of 3D-printed FDPs, in particular FDPs with w = 0.6 mm (108/197 µm vertical; 87/161 µm tangential). (4) Conclusions: 3D-printed zirconia FDPs showed conditionally comparable marginal and internal fit as their milled counterparts, but with slightly higher scattering. When fabricating thinner 3D-printed FDPs, additional support structures are mandatory to achieve clinically well-fitting restorations.

## 1. Introduction

The ability to maintain natural dentition throughout life has improved markedly in recent decades due to health awareness and advances in preventive and restorative dentistry. However, tooth losses are still a reality and a burden for patients. Periodontal disease, dental caries, trauma, and congenital absence continue to result in edentulous spaces, particularly in aging populations. Epidemiological data indicate that adults aged 35–44 have an average of 2.1 missing teeth but this number increases with older age [1]. Consequently, prosthetic rehabilitation remains an essential aspect of comprehensive dental care.

For edentulous spaces with adjacent teeth, fixed dental prostheses (FDPs) remain one of the most common treatment modalities. FDPs restore masticatory function, phonetics, and esthetics while preventing deleterious effects such as the drifting of adjacent teeth and overeruption of antagonists. Clinical success of FDPs depends primarily on the choice of restorative material and the fitting accuracy. Posterior tooth loss is associated with increased biomechanical demands on FDPs, which distinguishes posterior from anterior restorations and justifies a focused investigation on posterior FDPs. Occlusal forces in the premolar and molar regions are substantially higher and are frequently accompanied by torques arising from cusp morphology, eccentric contacts, and non-axial load direction [2]. From a clinical perspective, posterior FDPs are particularly sensitive to manufacturing-related inaccuracies and clinical success depends primarily on the choice of restorative material and the fitting accuracy. Poor marginal fit can lead to microleakage, cement dissolution, and subsequent biological complications, while inadequate mechanical performance can lead to catastrophic failure under functional loading.

Historically, FDP frameworks were fabricated from metal alloys using lost-wax casting techniques. Although metal–ceramic FDPs demonstrated excellent longevity with 10-year survival rates around 90%, their esthetic limitations have led to a paradigm shift toward all-ceramic materials [3]. The growing preference of patients for natural-appearing restorations has promoted the adoption of all-ceramic materials. Beyond its esthetic advantage, zirconia has become a preferred material for FDPs. 3 mol-% yttria-stabilized tetragonal zirconia polycrystal (3Y-TZP) has emerged as the material of choice for frameworks due to its high flexural strength (>900 MPa), excellent fracture toughness (7–10 MPa·m^1/2^), high survival rates up to 100% after 3 years and superior biocompatibility [4,5]. Another reason for the use of zirconia is the low plaque affinity and favorable wear behavior. In addition, the possibility of monolithic fabrication eliminates the risk of veneering ceramic chipping, which has been identified as a major complication of conventional metal–ceramic restorations. 3Y-TZP materials allow the fabrication of durable all-ceramic FDPs that combine both functional and mechanical reliability [6], and further modifications have made them applicable in a monolithic design, at least for posterior regions.

The widespread adoption of zirconia in prosthodontics was made possible by the advent of computer-aided design/computer-aided manufacturing (CAD/CAM) systems. Subtractive manufacturing, i.e., milling zirconia from pre-sintered blanks, is currently the gold standard [7,8,9,10]. In this workflow, oversized restorations are milled from partially sintered zirconia (“white-state”), followed by final sintering with 20–25% linear shrinkage to achieve full density [11]. However, milling is associated with certain limitations, such as high material waste (30–50% of the zirconia blank is discarded) [12], long milling times and the risk of material flaws caused by tool-induced stress at thin margins [13].

In response, additive manufacturing (AM), particularly 3D printing, has emerged as a promising technology in dentistry [10,14,15]. In 3D printing, restorations are built layer by layer from sliced CAD data, enabling complex geometries with less material waste [16]. Vat photopolymerization techniques, including stereolithography (SLA) and digital light processing (DLP), have been adapted to produce zirconia parts. In these methods, a photosensitive resin suspension containing zirconia nanoparticles is selectively cured. The “green-state” printed parts undergo debinding (removal of organic components) followed by high-temperature sintering to achieve fully dense ceramic restorations. In summary, 3D printing offers enhanced design freedom, material efficiency and shorter workflows [17,18,19].

Despite these theoretical benefits, the clinical adoption of 3D printing for definitive zirconia restorations is still lacking and most existing laboratory studies have focused on maximum single crowns, reporting clinically acceptable marginal fits [20,21]. Most research has addressed 3Y-TZP, which has been available for additive processing since the introduction of this technology and remains the strongest option. Materials with higher yttria content (4Y- or 5Y-PSZ) offer improved translucency but reduced mechanical performance. Nevertheless, 3D-printed zirconia materials exhibit, compared to zirconia used with the milling technology, increased porosity and consequently anisotropic behavior, slightly reduced bending strength, and inferior translucency [22,23]. Compared with single-tooth restorations, the fabrication of multi-unit prostheses remains more challenging. Increased span length and regions with increased material thickness (connectors and pontic) lead to a higher risk of distortions and dimensional deviations as well as the formation of porosity or cracks during the firing process. Currently, published evidence on the marginal and internal fit of three-unit posterior zirconia FDPs fabricated by additive manufacturing is limited to one theoretical study [24]. The question arises regarding whether 3D-printed three-unit FDPs can meet the requirements for clinical use before translating this technology into clinical routine [25].

The objectives of this study, therefore, were to evaluate whether three-unit posterior fixed dental prostheses fabricated by 3D printing can achieve clinically acceptable levels of fitting accuracy in comparison to milled counterparts and to investigate the influence of wall thickness and additional support optimization on this parameter. Additional sintering frames were introduced to possibly improve the fit of 3D-printed FDPs, as well to increase the bending stiffness of the 3D-printed objects during firing.

The null hypotheses were that there would be no significant difference in marginal or internal fit between 3D-printed and milled FDPs and that the accuracy of 3D-printed FDPs would not be influenced by an additional sintering frame.

## 2. Materials and Methods

### 2.1. Study and Sample Design

Since there was no reliable data on the fitting accuracy of minimally invasive 3D-printed FDPs in the posterior region and the effect of different support structures, the required sample size was estimated by the investigators and n = 10/test group was chosen. Therefore, the investigation was conducted as an exploratory study. The 3D-printed FDPs differed in wall thickness w (w = 0.6 mm/1.0 mm) and support structures (with/without a stiffening frame) during printing, cleaning, debinding, and presintering. Milled FDPs with w = 0.6 mm and no additional frame served as a control. Consequently, there were 5 test groups (Table 1).

**Figure 1:**
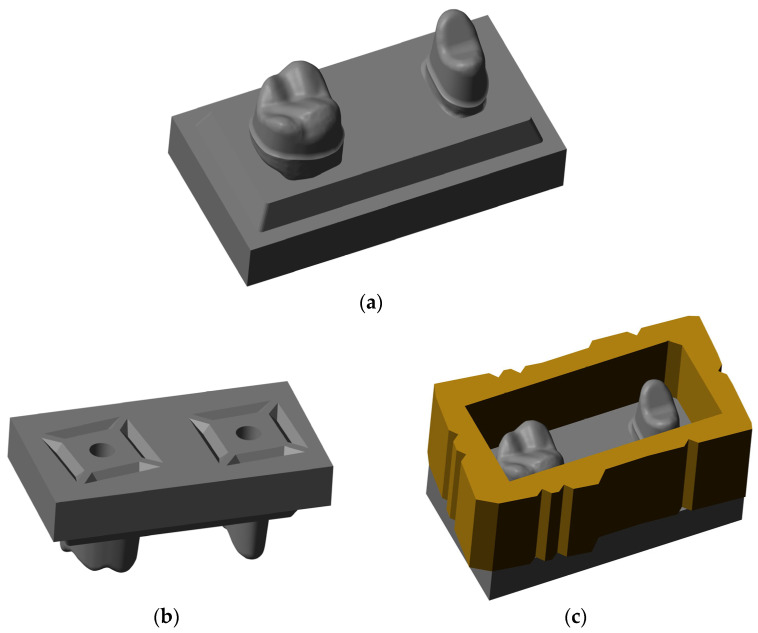
(**a**) Modified model scan, (**b**) the bottom side of the model base, and (**c**) the model with an additional frame to enable the embedding of FDPs cemented on the model.

Using a typodont maxillary model (ANA-01; Frasaco, Tettnang, Germany) missing the first molar, the second premolar and second molar were prepared with a 4° convergence angle and a chamfer finish line. The model was scanned (D2000; 3Shape, Copenhagen, Denmark) and the geometry modified as follows before model reproduction: dies were included in a planar rectangular base socket such that the insertion direction was identical to the vertical axis, the finishing lines were improved such that there were sharp edges at the boundaries of the prepared tooth surfaces (Figure 1a), and the bottom side of the model base included connection geometries for later fixation of the model (Figure 1b) (Geomagic Design X; 3D Systems, Rock Hill, SC, USA) (Figure 1). The final model geometries were produced from resin (Freeform Model grey 2.0; Detax, Ettlingen, Germany) using a 3D printer (Asiga Max UV; Asiga, Sydney, Australia). To enable embedding of the cemented FDPs later on, a form-congruent frame that could be placed on top of the model base was designed and 3D-printed from resin as well (Figure 1c).

3D-printed models were cleaned with isopropyl alcohol, light-cured (2 × 2000 flashed; Otoflash, NK Optik, Baierbrunn, Germany), and sandblasted (30 µm alumina particles, 0.1 MPa). All models were digitized (D2000/Convince 2015; 3Shape) and a fine mesh (50 µm triangle edge length) exported for the individual FDP design process. All scans were aligned with the original CAD data (Geomagic Design X; 3D Systems). Therefore, all scans were located at the same position and the vertical axis was the insertion axis for all FDPs in the following design process.

**Figure 2:**
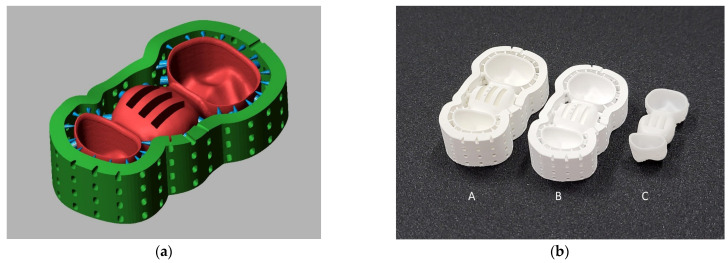
(**a**) Scaled CAD file of an FDP for 3D printing with basal slots and a stiffening frame; (**b**) images taken of a 3D-printed FDP after printing and cleaning (A, “green” state), after debinding and presintering (B, “white” state), and after final sintering (C, fully sintered FDP). Different numbers of dots on the basal surface served as an identifier for the individualized FDPs since 3 FDPs could be placed on the building platform during each 3D-printing job.

On the first model, an FDP template was designed with a constant abutment wall thickness of w = 0.6 mm and marginal gap widths of 30 µm and a cement gap width of 80 µm for both abutment teeth (Dental Designer 2018; 3shape). The design job was duplicated, the scan replaced by that of the second model, and the minimum wall thickness changed to 1.0 mm. After redefining the finishing lines, the complete design process (copied from the first model) could be repeated, resulting in a template for FDPs with w = 1.0 mm wall thickness. All following FDPs were designed as described above by copying the respective template, replacing the existing model scan by that of the current model, redefining the finishing lines, and automatically re-executing all design steps. Due to this procedure, all designed FDPs had identical pontic geometry and identical connectors. These FDP designs were only directly used for the test group with milled FDPs since a pilot test showed that very long debinding times would have been necessary with massive pontics. Therefore, the FDP pontic geometry was modified by implementation of three basal slots oriented in the mesio-distal direction (8 mm in length, 1.5 mm in width, and 1.4 mm spacing in the scaled state before 3D printing), allowing the use of a shorter firing protocol. The slots stopped at a 1 mm distance from the occlusal surface in the scaled design, corresponding to an approximate occlusal wall thickness above the slots of 0.75 mm after sintering. This modification was introduced in the pontic to reduce maximum material thickness, thereby facilitating binder removal and reducing thermal gradients during debinding and sintering. This approach promotes more homogeneous shrinkage behavior and lowers internal stresses associated with constrained densification of massive zirconia structures. This completed the design for 3D-printed FDPs without a stiffening frame, whereas for the remaining two groups, a stiffening frame was incorporated with the intention of potentially minimizing FDP distortions during the debinding and presintering process (Figure 2a). The used stiffening frame can reduce the buckling of thin crown margins and provides a high resistance against bending around the oro-buccal axis, thus having the potential to reduce distortions during the process and consequently improve the final fit of the restorations.

### 2.2. Sample Manufacturing

Each FDP was manufactured from 3Y-TZP, either by milling or 3D printing (Table 1). 3Y-TZP was selected because it represents the clinically established zirconia material for posterior FDPs, characterized by a high mean flexural strength (>1000 MPa) and Young’s modulus (210 GPa). Milled FDPs underwent an 8 h sintering protocol at up to 1500 °C in accordance with the manufacturer’s instructions. For the printed FDPs, preprocessing involved orientation selection, scaling, and xy-offset correction. The required parameters were assessed on a regular basis by 3D-printed zirconia calibration objects. Details about this process can be found in a previous publication [25]. Xy-offset correction offered by the ZiproS 3.1.106 software (AON) is done after slicing and discretization (40 µm grid for the used 3D printer) and consists of removing a given number of voxel layers from the object surface displayed in each slice. Thus, the nominal xy-offset can only be a multiple of the grid distance. In addition, the real offset is larger for surfaces not parallel to the grid orientation, with the maximum ($\sqrt{2}$ ∙ grid distance ∙ number of layers) found at 45° inclination. To circumvent this incremental and inhomogeneous xy-offset correction, scaling and surface offset were performed using a custom script (Matlab R2022a; Mathworks) that adjusted vertex coordinates while preserving mesh triangulation. The final data were transferred to the printer software (ZiproS, AON), occlusal supports were added and the slicing process was executed with a 50 µm layer thickness. 3D printing was performed under manufacturer-specified conditions at 26–28 °C. Postprocessing directly after printing included removal of occlusal support structures (stiffening the frame, if it existed, was not removed) and cleaning with an airbrush at 0.1 MPa using isopropanol. Debinding and presintering was carried out for 30 h at up to 1100 °C, after which the stiffening frame was removed in the presintered state. In this study, a staining process with staining liquids, which would take place after presintering during FDP fabrication in a clinical setting, was omitted. Final sintering was performed for 7 h 20 min at up to 1500 °C. Surfaces were sandblasted (50 µm alumina particles, 0.2 MPa) but not polished or glazed. Representative FDPs before and after sintering are shown in Figure 2b.

Basal slot surfaces were tribochemically pretreated (Rocatec Pre/Plus, 0.28 MPa), coated with a primer containing MDP and silane (Clearfill Ceramic Primer Plus; Kuraray, Tokyo, Japan), and filled with dual-curing composite resin (Rebilda DC; VOCO, Cuxhaven, Germany).

Adhesive cementation of the FDPs was performed with Panavia 21 TC (Kuraray Europe, Hattersheim am Main, Germany) using a universal testing machine (Z005; Zwick/Roell, Ulm, Germany) and an axial force of 200 N for 8 min. The applied load was selected to simulate controlled clinical seating forces reported for fixed dental prostheses and to ensure sufficient cement extrusion and complete seating, particularly for posterior multi-unit restorations [26]. The loading duration corresponded to the curing time of the used dual-curing resin cement provided by the manufacturer. In advance, internal FDP surfaces had been tribochemically silica-coated (Rocatec Pre: air-abrasion with 110 µm alumina particles for 10 s; Rocatec Plus: air-abrasion with silica-coated alumina particles for 12 s), followed by application of a primer (Clearfill Ceramic Primer plus; Kuraray Europe, Hattersheim, Germany) for 30 s. Following the 8 min loading period, restorations were stored for an additional 10 min at 37 °C in a heating cabinet.

### 2.3. Analyses of Marginal and Internal Fit

**Figure 3:**
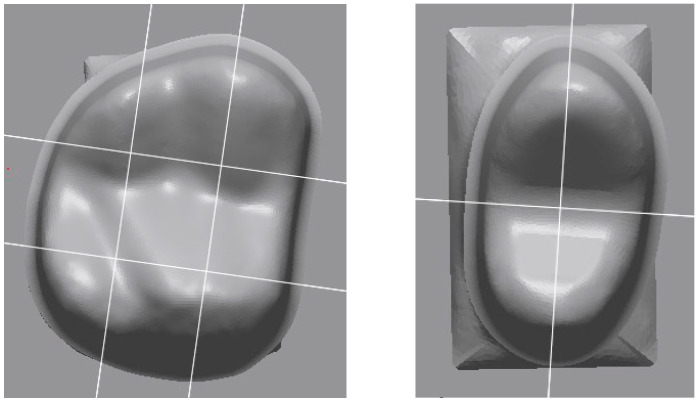
Planned positions of the sectioning planes for the molar and premolar abutment tooth.

Fitting accuracy was assessed using the sectioning method by one non-blinded operator (J.C.) since a previous study using the same method showed excellent repeatability of cement gap measurements and inter-rater reliability [10]. FDPs cemented on the respective resin models were sectioned at predefined locations, and the marginal fit (cement gap) was evaluated under a light microscope at specified measurement points. Sectioning sites were selected to be oriented approximately 90° to the abutment or crown surface and to intersect the cusp tips. For the molar, parallel sections were placed 3.0 mm apart in the oro-vestibular direction and 3.5 mm in the mesio-distal direction (Figure 3).

**Figure 4:**
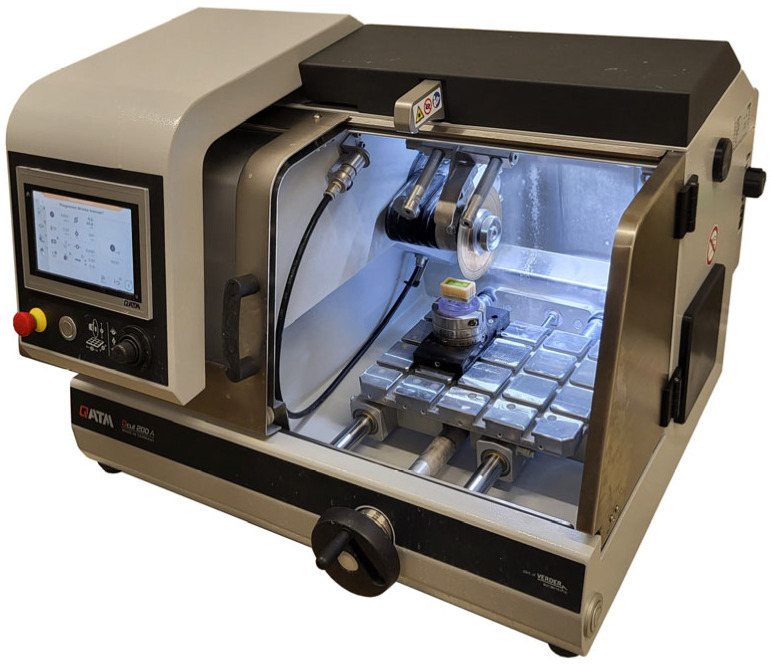
Sample placed in the precision cutting device for the first sectioning.

**Figure 5:**
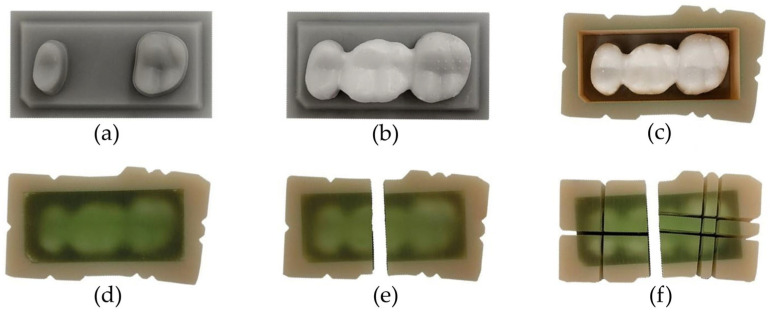
Embedding and sectioning process: (**a**) printed model, (**b**) model with adhesively cemented FDP, (**c**) model with FDP after placement of the embedding mold and (**d**) pouring with acrylic resin, (**e**) separation of the abutment teeth sections, and (**f**) completely sectioned model.

Before sectioning, the cemented FDPs were embedded in a casting mold using acrylic resin (Technovit 4071; Kulzer, Hanau, Germany) and sectioned mesio-distally and bucco-lingually using a precision cutting device (QCUT 200A; ATM Qness, Mammelzen, Germany) and a diamond-coated wheel (Diamond cut-off wheel B Ø125 × 0.5/5 × 12.7 mm; ATM Qness) under water cooling (Figure 4 and Figure 5).

**Figure 6:**
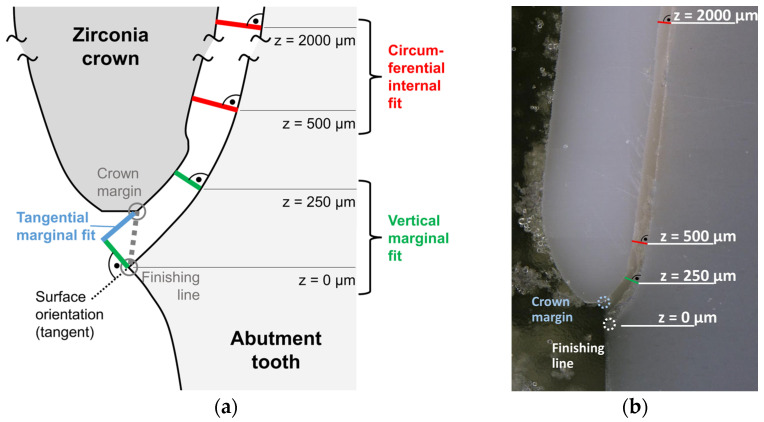
(**a**) Marginal and internal fit measurement points. (**b**) Microscopic analysis of marginal and internal fit (z: vertical distance).

**Figure 7:**
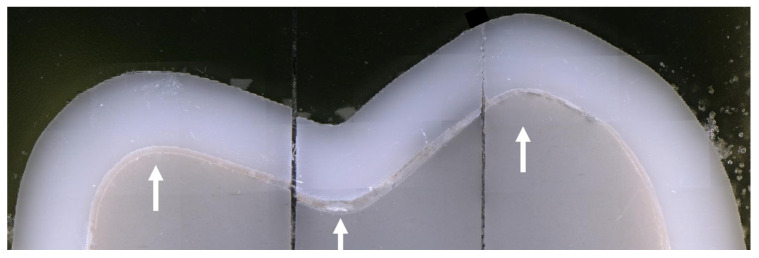
Microscopic analysis of occlusal fit; measurement points.

Cement gap measurements were performed using a digital microscope (Smartzoom 5; Carl Zeiss IQS, Oberkochen, Germany). For each plane, a sequence of images with 250× magnification was acquired and analyzed (ZEN core Version 3.2; Carl Zeiss). The assessment of fitting accuracy followed the approach described by Holmes et al. [27]. Marginal and internal adaptation were quantified at predefined locations. The position of the finishing line, which correlated with height z = 0 µm, was defined first. For each abutment tooth, measurement of cement gap widths was made perpendicular to the abutment tooth surface at heights z = 0 µm, 250 µm, 500 µm, and 2000 µm (Figure 6) and along the occlusal surface at the two cusps as well as the deepest point in between (Figure 7). For the mesio-distal section through the premolar without visible cusps, measurements were taken next to the edge of the oro-buccal section and at ±700 µm distance from that position. Tangential marginal fit was assessed as well (Figure 6a) with positive values indicating that the crown margin was located above the planned position. For final evaluation, cement gap widths were condensed as follows: (1) vertical marginal fit was associated with heights z = 0 µm and z = 250 µm, (2) circumferential internal fit was associated with heights z = 500 µm and z = 2000 µm (Figure 6), and (3) occlusal internal fit was associated with all measurements taken at the occlusal aspect (Figure 7).

### 2.4. Statistical Analysis

For each sample and each abutment tooth, mean and maximum values were computed for the condensed parameters defined above based on all associated data (all measurement locations and sections). Mean values for the complete FDP were gained by averaging mean values gained for the molar and premolar abutment tooth. Maximum values for the FDP were the largest values that existed in the originally measured data.

Descriptive statistics (for sample-based data) were performed using means and standard deviations (SDs) and minimum and maximum values as well as first, second (medians), and third quartiles. In addition, data were visualized by the use of boxplots. Since the data were not normally distributed (Shapiro–Wilk tests), differences in fitting accuracy between the test groups were analyzed using Kruskal–Wallis tests as well as post hoc Mann–Whitney U-tests. To address multiple pairwise testing, significance values were Bonferroni-adjusted (SPSS v28; IBM, Armonk, NY, USA). For possible differences in fit between molar and premolar abutment teeth, Wilcoxon signed-rank tests were applied. The significance level for all tests was set to α = 0.05.

## 3. Results

The placement of all FDPs on their respective models before cementation revealed no macroscopic problems with regard to fitting accuracy. Without the adhesive resin cement, all FDPs were seated in the intended final position and no large gaps could be seen during visual inspection.

**Figure 8:**
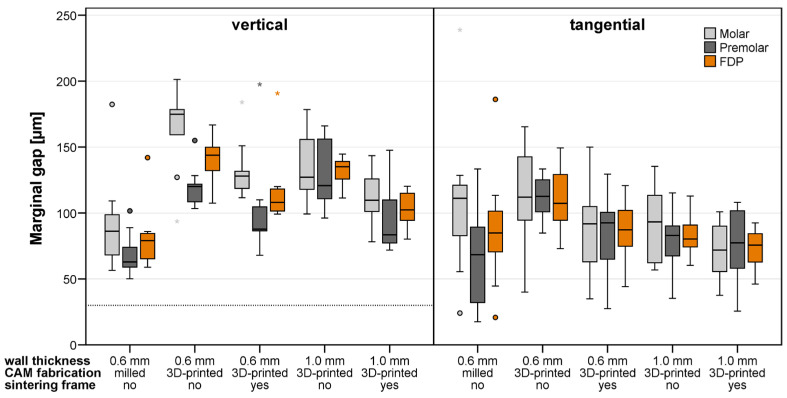
Vertical and tangential marginal fit of milled and 3D-printed FDPs quantified by mean cement gap widths measured at the two abutment teeth and the whole FDP. The dotted line marks the nominal gap width defined during FDP design. Circles and asterixes indicate mild and extreme outliers, defined as values lying more than 1.5 and 3.0 times the interquartile range away from the box.

**Figure 9:**
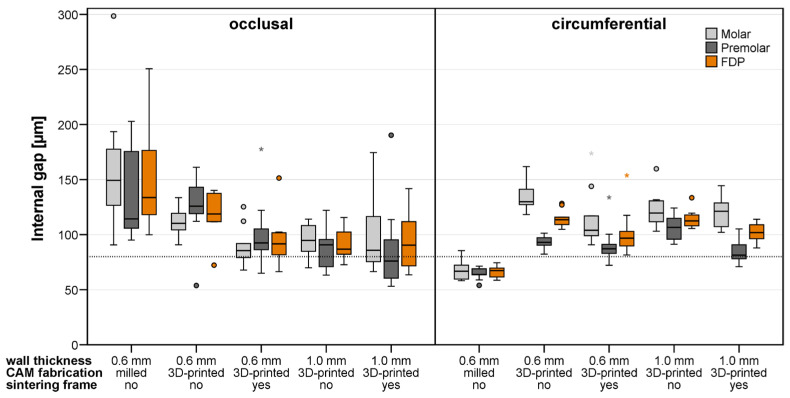
Occlusal and circumferential internal fit of milled and 3D-printed FDPs quantified by mean cement gap widths measured at the two abutment teeth and the whole FDP. The dotted line marks the nominal gap width defined during FDP design. Circles and asterixes indicate mild and extreme outliers, defined as values lying more than 1.5 and 3.0 times the interquartile range away from the box.

The mean and maximum gap widths determined for the FDPs of the different test groups differing in fabrication, wall thickness w, and stiffening frame are summarized in Table 2. The boxplots in Figure 8 and Figure 9 also provide, besides the results for the complete FDPs, the mean fitting parameters assessed for the single abutment teeth.

Overall, the marginal and internal gap widths observed in this study were predominantly within ranges that are generally considered clinically acceptable. Mean marginal gap values for most groups were below or close to commonly reported clinical thresholds of approximately 100–120 µm, whereas maximum gap values occasionally exceeded these limits, particularly in 3D-printed FDPs without a stiffening frame. Internal gap widths were largely within the range proposed as acceptable for occlusal and circumferential fit (<250–300 µm), although increased variability was observed in thin-walled 3D-printed FDPs without additional support structures.

### 3.1. Vertical Marginal Fit

Vertical marginal fit results revealed notable differences among the tested manufacturing methods and wall thickness configurations (Kruskal–Wallis tests, *p* < 0.001 for both mean and maximum vertical marginal gap widths). Pairwise comparisons demonstrated that the milled FDPs with w = 0.6 mm showed significantly lower mean marginal gap widths (median: 79 µm) compared with all printed groups (adjusted *p*-values, *p* < 0.05) and remained within commonly accepted clinical limits. With the exception of 3D-printed FDPs with w = 1.0 mm and a stiffening frame (*p* = 0.09), the same was true for the maximum gap widths of milled FDPs (median: 127 µm, *p* < 0.02 when compared to the remaining groups). The worst fit was associated with 3D-printed FDPs without a stiffening frame (median values: 144 µm for mean and 284 µm for maximum gap width for FDPs with w = 0.6 mm, and 135 µm for mean and 248 µm for maximum gap width for FDPs with w = 1.0 mm) as the values frequently exceeded clinically acceptable thresholds. The implementation of a stiffening frame could improve the vertical marginal fit, but not all pairwise tests indicated significant differences after Bonferroni correction (w = 0.6 mm: *p* = 0.07 for mean and *p* = 0.02 for maximum gap width; w = 1.0 mm: *p* < 0.01 for mean and *p* = 0.15 for maximum gap width). The overall trend indicated enhanced dimensional stability and clinically more acceptable marginal adaptation when additional support structures were applied.

### 3.2. Tangential Marginal Fit

Mean and maximum tangential marginal fit values were positive for all test groups, indicating that either the final crown positions were higher than intended or crown margins were too short. Tangential marginal fit analysis revealed no statistically significant differences among the evaluated manufacturing methods and wall thickness configurations (Kruskal–Wallis test, *p* = 0.051 for mean values and *p* = 0.515 for maximum values) and the values generally remained within ranges considered clinically acceptable. The printed groups demonstrated marginal adaptation conditionally comparable to the milled group, with median mean gap widths ranging from 76 to 87 µm and maximum gap widths from 161 to 180 µm. In contrast, FDPs with w = 0.6 mm and without a stiffening frame showed the poorest fit (median value: 107 µm and maximum gap of 198 µm).

### 3.3. Occlusal Internal Fit

Occlusal internal fit analysis revealed significant differences among the tested groups (*p* < 0.001 for mean values and *p* = 0.03 for maximal occlusal internal gap widths). Pairwise comparisons indicated significantly higher mean internal gap widths (median: 134 µm) of the milled group compared to all printed groups (*p* < 0.02), although these values remained within clinically acceptable ranges reported for occlusal fit. Except for 3D-printed FDPs with w = 0.6 mm and no stiffening frame, all printed groups demonstrated internal median gap widths of 87–92 µm (which were only slightly above the nominal cement gap width of 80 µm) and maximum gap widths of 151–163 µm. The implementation of a stiffening frame did not significantly improve the occlusal internal fit.

### 3.4. Circumferential Internal Fit

Circumferential internal fit analysis demonstrated significant differences among the tested manufacturing methods and wall thickness configurations (*p* < 0.001 for both mean and maximum gap widths). The milled FDP group showed significantly lower gap widths (median: 67 µm, maximum: 107 µm) lying below the nominal cement gap width compared with all printed groups (*p* < 0.001) that showed gap widths above the nominal cement gap width and within clinically acceptable limits. Implementation of a stiffening frame significantly improved circumferential internal fit (w = 1 mm: *p* = 0.05), with FDPs without a stiffening frame showing median gap widths of 113 µm (mean) and 230 µm (maximum) versus 102 µm (mean) and 208 µm (maximum) for FDPs with a stiffening frame, resulting in values closer to the nominal cement gap and improved clinical acceptability. Among the printed groups, FDPs with a wall thickness of 0.6 mm combined with a stiffening frame revealed the best circumferential internal fit (median: 97 µm, maximum: 175 µm, *p* < 0.03).

### 3.5. Differences Between the Abutment Teeth

A non-parametric comparison for paired samples (Wilcoxon signed-rank test) was performed to assess overall differences in the fitting accuracy observed at the molar and premolar abutment tooth. As can be seen from Figure 8 and Figure 9, higher or equivalent cement gap widths were found at the molar abutment teeth when compared to the premolar abutment teeth at all investigated locations. These differences were significant for the vertical marginal fit and circumferential internal fit when mean gap widths were analyzed (*p* < 0.001) and for all measures when maximum gap widths were of interest (*p* < 0.001).

## 4. Discussion

The present study addresses a relevant gap in the current literature on additively manufactured zirconia restorations. While the marginal and internal fit of 3D-printed single crowns has been investigated in several in vitro studies, evidence on multi-unit FDPs, especially three-unit posterior FDPs fabricated from 3Y-TZP, is scarce [21].To the authors’ knowledge, this is the first experimental study to systematically evaluate the fit accuracy of cemented FDPs produced by additive manufacturing and to directly compare them with a clinically established milling workflow under standardized conditions. Moreover, the present work investigates the influence of wall thickness and the implementation of a temporary stiffening frame during debinding and sintering, aiming to mitigate distortion-related inaccuracies that are specific to additively manufactured multi-unit restorations. Against this background, the present findings did demonstrate that for 3D-printed zirconia FDPs, comparable marginal and internal fit to milled counterparts lying below thresholds for clinical recommendation could be achieved. The rejection of both null hypotheses underscores that the fitting accuracy of 3D-printed zirconia FDPs is influenced by design- and process-related parameters, which must be considered for successful clinical translation.

The clinical standard for fabrication of zirconia restorations is subtractive manufacturing. This technique has been shown to reliably produce restorations with low marginal discrepancies [28]. A review by Svanborg et al. [29] reported mean marginal and internal gaps of 83 µm and 111 µm, respectively. In the present study, the median values for vertical/tangential marginal gap widths of milled FDPs were 79/85 µm when addressing mean gap widths and 127/171 µm when addressing the maximum gap width of each FDP. This was clearly above the nominal marginal cement gap width of 30 µm used during the design process. Abutment crown cavities of milled FDPs tended to be a little bit too narrow since median circumferential gap widths lay 13 µm below the nominal value of 80 µm. This may have led to those FDPs not completely reaching the intended end position, i.e., the median internal occlusal gap width was 54 µm above the nominal value. Especially when marginal fit is assessed, the specification of maximum gap widths is valuable information that should be considered in order to better interpret the results. A crown with perfect fit along 90% of the margin and a large defect along the remaining 10% may be considered not sufficient for clinical use by a dentist. Nevertheless, despite acknowledging the presence of variability in gap widths within single restorations, most studies present only mean gap openings and only a limited number also provide maximum gap widths.

The observed combination of undersized circumferential internal gaps and oversized occlusal gaps in the milled FDP group can be explained by mechanisms caused by slight imperfections and inaccuracies inherent to subtractive CAD/CAM fabrication. This effect has been reported for zirconia crowns and FDPs and may lead to increased friction and premature binding surfaces along the circumferential walls during seating if internal surfaces are not manually adapted [30,31]. In these investigations, the fit of milled FDPs was a little bit tighter than intended, i.e., the circumferential cement gap was below the target value in the final position even though the FDP had not yet reached the planned vertical position.

Memarian et al. [32] evaluated three-unit posterior zirconia FDPs fabricated with the same milling unit (Cercon Brain Expert) and found occlusal gaps of 98.7 µm and marginal discrepancies of 116.9 µm, though their definition of “marginal fit” remained unclear, which remains a frequent limitation in the literature. Other authors [33,34] reported mean marginal gaps of 106 µm and occlusal gaps of 248 µm for conventionally fabricated three-unit zirconia FDPs.

Clinically, marginal gaps below 100 µm are generally considered acceptable [10], although thresholds of <120 µm [32] and <200 µm [34,35] have also been proposed. For occlusal fit, values of <250 µm or <300 µm have been suggested [36,37].

In this study, lower mean marginal gap widths (median value < 108 µm) and internal gaps ranging from 102 to 92 µm were observed for 3D-printed FDPs with a sintering frame. The results indicate that 3D-printed zirconia FDPs can achieve marginal and internal fit conditionally comparable to milled restorations, provided that adequate wall thickness and support structures are implemented. In contrast, 3D-printed FDPs with reduced wall thickness (0.6 mm) and without a sintering frame showed higher gap values and greater variability, with several outliers reaching 287 µm. This finding suggests increased susceptibility to deformation during debinding and sintering when no reinforcement is applied. The clinical relevance of wall thickness is further supported by previous investigations on zirconia restorations, which demonstrated that reduced zirconia thickness markedly increases susceptibility to clinically relevant changes in material behavior, including optical properties, underscoring the need for sufficient structural thickness in zirconia-based restorations [38]. In the present context, it should be considered that massive pontic geometries require extended debinding and presintering cycles, which may in turn increase the risk of thermal-induced defects and prolong the overall fabrication time, which is higher than milling time. However, shorter debinding protocols have been reported for additive systems using high wall thicknesses and massive pontics [39]. These approaches were associated with higher incidence of cracks, and it remains uncertain whether ultra-fast procedures can fulfill the material requirements for dental zirconia. Previous studies on 3D-printed dental materials have shown pronounced material- and process-dependent differences in mechanical behavior, highlighting that design and processing strategies must be adapted to the specific manufacturing technology rather than transferred directly from conventional workflows [40].

In the current study, basal modifications of the pontic were therefore implemented to reduce material volume and firing duration. Consequently, future studies should focus on hollow pontic designs that more effectively decrease material mass, facilitate binder removal and enable shorter debinding protocols.

The stiffening frame was intended as a temporary reinforcement during debinding and presintering, i.e., in the processing phase during which the material exhibits limited strength and is most susceptible to creep-like deformation and warpage. From a structural mechanics perspective, the frame increases the effective bending stiffness of the FDP, in particular around the oro-buccal axis, thus reducing deflections induced by gravity, handling, and stresses originating from non-uniform shrinkage or thermal gradients within the FDP. The effect is expected to be most pronounced in long-span geometries with thin walls, where small deformations in the green/white state can translate into clinically relevant marginal discrepancies after sintering.

Previous studies on 3D-printed dental materials have shown pronounced material- and process-dependent differences in mechanical behavior, highlighting that design and processing strategies must be adapted to the specific manufacturing technology rather than transferred directly from conventional workflows.

When interpreting the present results from a clinical perspective, it is essential to distinguish between mean and maximum marginal discrepancies. Although the mean marginal gap values of several 3D-printed FDP groups were within ranges commonly considered clinically acceptable, maximum marginal gaps reached values of 280–290 µm in thin-walled FDPs fabricated without a stiffening frame. Accordingly, these findings cannot be regarded as clinically equivalent to milled FDPs. In contrast, 3D-printed FDPs fabricated with a stiffening frame showed lower maximum marginal gaps, approaching the range reported for conventionally fabricated multi-unit zirconia restorations. These findings indicate that clinical comparability to milled FDPs is configuration-dependent and requires strict control of design and processing parameters.

Lüchtenborg et al. [24] evaluated the manufacturing accuracy of four-unit monolithic zirconia FDPs fabricated using different additive manufacturing techniques. Using two DLP printers, internal deviations of up to 288 µm and marginal discrepancies of up to 389 µm were reported. SLA-produced FDPs showed internal deviations of 68 µm and marginal gaps of 70 µm. Conventionally milled controls demonstrated significantly superior accuracy compared to all additive approaches.

Elsayed et al. [41] compared the marginal adaptation of anatomical premolar crowns fabricated by 3D printing and milling from 3Y-TZP. Cemented crowns revealed marginal gaps of 45 µm for 3D-printed and 19 µm for milled restorations, with the latter showing significantly smaller values (*p* = 0.011, significance level *p* ≤ 0.05). The study also demonstrated that cementation negatively affected marginal fit.

Other authors [20,42,43] investigated premolar crowns fabricated additively and subtractively, showing mean marginal discrepancies of 100 µm for 3D-printed and 60 µm for milled crowns. Others examined fully anatomical molar crowns fabricated with two SLA printers. Marginal gaps of 93 µm and 109 µm and occlusal discrepancies of 210 µm and 149 µm were reported.

In this study, fit accuracy was assessed using the cross-section method, which allowed direct measurement of the cement gap after standardized cementation. Although slight deviations in sectioning and occasional edge chipping may have occurred, the method ensured stable specimen geometry and clear identification of measurement areas, an advantage over silicone replica techniques.

One strength of this study was the use of a standardized cementation protocol with a universal testing machine, ensuring strictly controlled and reproducible load application with constant force vectors across specimens. In addition to the general comparison between manufacturing methods, the present results demonstrated that the premolar and molar abutment teeth showed different fitting characteristics across all tested groups. This pattern was observed for both milled and 3D-printed FDPs, suggesting that tooth-specific anatomical and geometric configurations influence the fit regardless of the fabrication method. Molars present a larger and more complex internal geometries and are likely to impede uniform cement extrusion during cementation, potentially resulting in increased gaps, especially for the occlusal and circumferential internal fit. As stated at the beginning of the Results Section, there was no visible misfit of the FDPs when placing them on the respective models without cement. However, final FDP positions after cementation were generally a little bit higher than intended, which can be attributed to the adhesive cementation that was essential for study-related subsequent cross-sectioning. This highlights that FDP cementation is a crucial part when evaluating FDP fitting accuracy. In a clinical setting, various (conventional) cements with different characteristics can be used. The type of cement may also have influenced the fit of FDPs, particularly with regard to effective cement extrusion. These tooth-specific effects suggest that differences in abutment morphology may be a relevant reason for the observed variability and must be considered when interpreting fit results.

The milled control group represented a well-established, highly standardized workflow with minimal sources of error. In contrast, additive manufacturing remains relatively new and prone to inaccuracies during printing, cleaning and sintering [44,45,46]. The FDP geometries were defined in a way that avoided sharp internal angles and undercuts, eliminating the need for radius compensation of the milled group during the milling process, since the inability of milling tools to reproduce fine internal radii may lead to excessive internal adjustments or increased cement gaps. In this case, the observed differences in fitting accuracy cannot be attributed to design-related limitations of the milling workflow but should rather be interpreted as inherent outcomes of the respective fabrication techniques and subsequent processing steps.

The comparison between milled and 3D-printed FDPs reflects technology-specific design and processing requirements rather than identical geometries. While 3D-printed FDPs required modifications such as basal slots to reduce material thickness of the pontic, temporary reinforcement, and customized scaling, milled FDPs followed a standardized subtractive workflow. Accordingly, the present comparison reflects two optimized and clinically relevant fabrication workflows.

The present in vitro study has several limitations that should be considered when interpreting the results. From the methodological perspective, the study was performed on standardized resin models under highly controlled laboratory conditions. While this approach ensured reproducibility and minimized confounding variables, it does not fully reflect the clinical situation, where variations in tooth preparation geometry, finish line quality, and abutment mobility may influence restoration fit. In addition, all FDPs were fabricated and post-processed by a single operator, which reduced operator-dependent variability but limited generalizability to routine laboratory workflows involving multiple technicians.

Material- and process-related limitations arise from the focus on a single 3Y-TZP material and one additive manufacturing system. Although 3Y-TZP remains the material of choice for posterior FDPs due to its superior mechanical properties, other zirconia compositions (4Y- or 5Y-TZP) and alternative printing technologies may show different distortion behaviors during debinding and sintering. Furthermore, the modified pontic design and omission of staining and glazing steps were chosen to standardize processing and shorten firing cycles, but these design and postprocessing choices may influence fit, surface quality, and long-term performance in a clinical setting.

Adhesive cementation under controlled axial loading was chosen to ensure standardized and reproducible conditions but led to increased occlusal internal gaps. The lack of a non-cemented control group and the use of only one adhesive cement limit the ability to separate manufacturing-related fit characteristics from cementation-induced seating effects.

Further research should therefore focus on validating these findings under more clinically relevant conditions. This includes the investigation of different zirconia generations and 3D-printing systems, optimization of support and reinforcement strategies for various FDP geometries, and systematic evaluation of alternative pontic designs aimed at reducing material volume while preserving mechanical stability. In addition, studies incorporating different cementation protocols and ultimately prospective clinical trials are required to assess the long-term performance and survival of 3D-printed multi-unit FDPs.

## 5. Conclusions

Within the limitations of this in vitro study, three-unit posterior FDPs fabricated from 3Y-TZP by 3D printing showed marginal and internal cement gap widths conditionally comparable to those of milled FDPs, provided that appropriate design- and process-related measures were applied. In 3D-printed FDPs, fitting accuracy was significantly influenced by wall thickness and the use of a temporary stiffening frame during debinding and presintering. Thin-walled restorations fabricated without a stiffening frame showed the largest marginal discrepancies and highest variability, whereas the stiffening frame improved vertical marginal and circumferential internal fit. From a clinical perspective, 3D-printed three-unit zirconia FDPs may represent a viable alternative to milling in posterior regions, provided that strict process control, adequate wall thickness, and temporary reinforcement during thermal processing are ensured. Future clinical trials are needed to verify these outcomes and will be a milestone for translating prospective studies evaluating the long-term survival of 3D-printed multi-unit zirconia.

## Figures and Tables

**Table 1:** Overview of the five study groups as well as involved materials and devices.

Fabrication	w [mm]	Stiffening Frame	Zirconia/CAM Device	Furnace
milled	0.6	no	Cercon ht/Cercon Brain Expert (Dentsply Sirona, Hanau, Germany)	Cercon heat plus (Degudent, Hanau, Germany)
3D-prin-ted	0.6	no	InniCera BCM W1000/ZIPRO-D Dental (AON, Seoul, Republic of Korea)	Presintering: ZIRFUR (AON, Seoul, Republic of Korea) Final Sintering: SINTRA PRO/120 zrf (Shenpaz Dental, Migdal HaEmek, Israel)
		yes		
	1.0	no		
		yes		

**Table 2:** Mean and maximum gap width of milled and 3D-printed FDPs. w: wall thickness, Frame: stiffening frame structure during printing and firing, mean: mean value, SD: standard deviation, min: minimum, Q1: first quartile, Q2: median, Q3: third quartile, and max: maximum.

		Fabrication	w	Frame	Mean Cement Gap Width [µm]							Maximum Cement Gap Width [µm]						
			[mm]		Mean	SD	Min	Q1	Q2	Q3	Max	Mean	SD	Min	Q1	Q2	Q3	Max
M a r g i n a l f i t	Vertical	milled	0.6	no	81	23	59	65	79	85	142	153	69	104	119	127	175	337
		3D-printed	0.6	no	142	17	108	132	144	150	167	292	44	234	261	284	313	392
			0.6	yes	116	27	99	102	108	118	191	214	46	179	183	197	235	327
			1.0	no	132	10	111	126	135	139	145	243	36	184	214	248	276	284
			1.0	yes	103	12	80	94	102	115	120	200	32	155	172	196	225	252
	Tangential	milled	0.6	no	87	44	21	71	85	101	186	173	65	42	154	171	182	306
		3D-printed	0.6	no	111	27	73	95	107	129	149	203	54	143	156	198	225	299
			0.6	yes	86	26	44	75	87	102	121	174	53	87	135	161	223	249
			1.0	no	85	17	60	74	80	91	113	188	59	99	143	180	246	280
			1.0	yes	74	14	46	63	76	84	93	158	30	106	132	166	181	193
I n t e r n a l f i t	Occlusal	milled	0.6	no	147	45	100	118	134	177	251	203	55	134	169	198	221	334
		3D-printed	0.6	no	119	20	72	112	119	138	140	195	22	155	186	192	214	225
			0.6	yes	95	23	66	82	92	102	151	157	40	99	126	151	173	225
			1.0	no	90	13	73	82	87	102	116	160	21	124	145	162	169	196
			1.0	yes	94	25	64	72	90	112	142	167	46	101	129	163	206	231
	Circumferential	milled	0.6	no	66	5	59	62	67	70	75	122	36	78	97	107	165	170
		3D-printed	0.6	no	114	8	105	109	114	116	128	265	40	210	232	259	286	346
			0.6	yes	102	21	82	90	97	103	154	187	41	150	163	175	200	291
			1.0	no	114	8	106	108	113	118	134	224	31	171	202	230	237	268
			1.0	yes	102	8	88	96	102	109	114	209	29	163	188	208	226	254

## Data Availability

The original contributions presented in this study are included in the article. Further inquiries can be directed to the corresponding author.
